# Role of Src and Cortactin in Pemphigus Skin Blistering

**DOI:** 10.3389/fimmu.2019.00626

**Published:** 2019-04-04

**Authors:** Daniela Kugelmann, Vera Rötzer, Elias Walter, Desalegn Tadesse Egu, Michael Tobias Fuchs, Franziska Vielmuth, Hilda Vargas-Robles, Michael Schnoor, Michael Hertl, Rüdiger Eming, Klemens Rottner, Ansgar Schmidt, Volker Spindler, Jens Waschke

**Affiliations:** ^1^Chair of Vegetative Anatomy, Institute of Anatomy, Medical Faculty, Ludwig-Maximilians-Universität München, Munich, Germany; ^2^Department of Molecular Biomedicine, Cinvestav-IPN, Mexico City, Mexico; ^3^Department of Dermatology and Allergology, Philipps-Universität Marburg, Marburg, Germany; ^4^Division of Molecular Cell Biology, Zoological Institute, Technische Universität Braunschweig, Braunschweig, Germany; ^5^Department of Cell Biology, Helmholtz Centre for Infection Research, Braunschweig, Germany; ^6^Instiute of Pathology, Philipps-Universität Marburg, Marburg, Germany; ^7^Department of Biomedicine, University of Basel, Basel, Switzerland

**Keywords:** src, cortactin, adhesion, skin blistering diseases, pemphigus vulgaris (PV)

## Abstract

Autoantibodies against desmoglein (Dsg) 1 and Dsg3 primarily cause blister formation in the autoimmune disease pemphigus vulgaris (PV). Src was proposed to contribute to loss of keratinocyte cohesion. However, the role and underlying mechanisms are unclear and were studied here. In keratinocytes, cell cohesion in response to autoantibodies was reduced in Src-dependent manner by two patient-derived PV-IgG fractions as well as by AK23 but not by a third PV-IgG fraction, although Src was activated by all autoantibodies. Loss of cell cohesion was progredient in a timeframe of 24 h and AK23, similar to PV-IgG, interfered with reconstitution of cell cohesion after Ca^2+^-switch, indicating that the autoantibodies also interfered with desmosome assembly. Dsg3 co-localized along cell contacts and interacted with the Src substrate cortactin. In keratinocytes isolated from cortactin-deficient mice, cell adhesion was impaired and Src-mediated inhibition of AK23-induced loss of cell cohesion for 24 h was significantly reduced compared to wild-type (wt) cells. Similarly, AK23 impaired reconstitution of cell adhesion was Src-dependent only in the presence of cortactin. Likewise, Src inhibition significantly reduced AK23-induced skin blistering in wt but not cortactin-deficient mice. These data suggest that the Src-mediated long-term effects of AK23 on loss of cell cohesion and skin blistering are dependent on cortactin-mediated desmosome assembly. However, in human epidermis PV-IgG-induced skin blistering and ultrastructural alterations of desmosomes were not affected by Src inhibition, indicating that Src may not be critical for skin blistering in intact human skin, at least when high levels of autoantibodies targeting Dsg1 are present.

## Introduction

Pemphigus vulgaris (PV) is a severe autoimmune skin blistering disease. Patients suffer from mucocutaneous erosions and blisters caused by autoantibody-induced acantholysis (1, 2). It is accepted that loss of keratinocyte cohesion is primarily caused by autoantibodies directed against the desmosomal cadherins desmoglein 3 (Dsg3) and Dsg1 (3). Autoantibodies were proposed to directly interfere with trans-interaction of Dsg3 and to require cellular signaling mechanisms to induce loss of cell adhesion (2). Pemphigus autoantibodies modulate the activity of signaling pathways such as Ca^2+^ -influx, protein kinase C (PKC), p38 mitogen-activated protein kinase (p38MAPK) and sarcoma-associated kinase (Src), and interfere with the turnover of desmosomal components (2). It was shown that Src can be activated by PV-IgG containing antibodies against Dsg3 as well as by AK23, which constitutes a Dsg3-specific monoclonal autoantibody from a pemphigus mouse model (4, 5). However, the role of Src in the loss of keratinocyte cohesion and the underlying mechanisms are not fully understood. Src is a signal-transducing non-receptor protein kinase which is involved in several signaling pathways (6). It is enriched in various cancer diseases and plays an important role in metastasis, cell migration and motility as well as cell survival and proliferation (6–9). Furthermore, Src is located at adherens junctions in various cell types (10), and the inhibition of Src-kinase activity is proposed to stabilize cadherin dependent cell-cell contacts (11). In previous studies we showed that PV-IgG-mediated loss of keratinocyte cohesion can be abrogated by Src inhibition (4), and that the activity of Src in combination with E-cadherin is necessary for the cytoskeletal anchorage of Dsg3 (12). Moreover, Dsg3 forms a complex with Src and regulates its activity (12, 13). All these findings led to the hypothesis that Dsg3 organizes cell contacts coordinating cell adhesion with signaling responses required for cellular behavior (14). Thus, we speculated that Src could be a key regulator of desmosomal adhesion in PV pathogenesis. In this context, it was demonstrated that autoantibodies from pemphigus patients caused Src-dependent phosphorylation of Pkp3 paralleled by Dsg3 translocation to the cytoplasm and destabilization of cell adhesion (15). Since we observed that Src is required for desmosome assembly, which is regulated by actin-binding proteins such as adducin (12, 16), we focused on the actin binding protein cortactin, which was identified as a major substrate for Src (17). Among many other functions, phosphorylation of cortactin is crucial for cadherin-mediated intercellular adhesion strength (18). In the work presented here, we show for the first time that cortactin regulates reconstitution of cell adhesion in pemphigus and provide new insights into the role and function of Src in PV.

## Materials and Methods

### Cell Culture

The immortalized human keratinocyte cell line HaCaT was cultured in Dulbecco's Modified Eagle Medium (DMEM) (Life Technologies, Carlsbad, CA) supplemented with 10% FCS (Biochrom, Berlin, Germany), 50 U/ml penicillin and 50 g/ml streptomycin (both AppliChem, Darmstadt, Germany) in a humidified atmosphere of 5% CO_2_ at 37°C.

### Preparation of Mouse Keratinocytes

Murine keratinocytes from the epidermis of newborn cortactin-deficient (CTTN^−/−^) and cortactin wildtype (CTTN^+/+^) mice were isolated and immortalized according to the literature for preparation of mouse keratinocytes (19–21). In brief, the skin was taken and incubated for 16 h in 2.4 U/ml dispase II in PBS supplemented with Gentamicin/AmphotericinB (CELLnTEC, Bern, Switzerland) at 4°C. After separating the dermis and epidermis, the epidermis was incubated for 20 min with accutase (CELLnTEC) at room temperature, in order to dissociate the cells. Mouse keratinocytes were resuspended and then grown in complete FAD medium (0.05 mM CaCl) on collagen I-coated culture dishes (rat tail; BD Bioscience, New Jersey, USA). The cells were cultivated in a humidified atmosphere containing 5% CO_2_ at 35°C. After reaching confluence, cells were switched to 1.2 mM Ca^2+^ and used for experiments after 48 h.

### Test Reagents, Antibodies, and Purification of PV-IgG Fractions

The Src-inhibitor PP2 (Calbiochem, Darmstadt, Germany) was used at 10 μM for the respective time periods. For short time-incubations PP2 was preincubated for 2 h. The following commercial primary antibodies were used: anti- cortactin (clone 4F-11; Milipore), anti-phospho-cortactin tyr421 (Milipore), anti-alpha-tubulin (Abcam), anti-desmoplakin (DP) (Abcam), anti-desmoglein 3 (Dsg3) (Clone H154, Santa Cruz), anti-Src (Clone 32G6, Cell Signaling), anti-phospho-Src Tyr 416 (Cell Signaling), anti-plakophilin 3 (Pkp3) (Progen), anti-phospho-plakophilin Tyr 195 (kindly provided by Ansgar Schmidt, Marburg, Germany). Filamentous actin (F-actin) was visualized using an Alexa Fluor^TM^ 488 phalloidin dye (Life Technologies). The corresponding secondary antibodies for immunofluorescence analysis were purchased from Dianova (Hamburg). AK23, a monoclonal pathogenic antibody, derived from a pemphigus mouse model, was purchased from Biozol (Eching, Germany) and used at 75 μg/ml. PV-IgG (all including autoantibodies against both Dsg1 and Dsg3) and IgG fractions pooled from three healthy donors (c-IgG) were purified as described previously (22). The autoantibody profiles were determined using enzyme-linked immunosorbent assay (ELISA), (Euroimmun, Luebeck, Germany). ELISA scores of antibodies against Dsg1 and Dsg3 were determinded before purification (cut-off value: 20 U/ml). The scores are shown in Table 1. All patients had mucocutaneous involvement. Patients and donors gave written consent for research use. A positive vote of the Ethics Committee from the Medical Faculty of the University of Marburg was given.

**Table 1:** Antibody profiles of pemphigus vulgaris patients' IgG fractions as determined by ELISA for Dsg1 or Dsg3, respectively, and clinical phenotype.

**ELISA**	**Dsg1 (U/ml)**	**Dsg3 (U/ml)**	
PV1-IgG	375	11.55	Mucocutaneous PV
PV2-IgG	212.27	181.44	Mucocutaneous PV
PV3-IgG	101.18	106.72	Mucocutaneous PV
PV4-IgG	5542	711	Mucocutaneous PV

### Western Blotting

Cells were washed with PBS, lysed with SDS-lysis buffer (25 mmol/l HEPES, 25 mmol/l NaF and 1% SDS, pH 7.4) and sonicated on ice. Protein amounts were determined using the Pierce^TM^ BCA Protein Assay Kit (Thermo Fisher, USA). Cell lysates were mixed with laemmli buffer containing 50 mM dithiothreitol. Electrophoresis and western blotting were performed according to standard procedures. Membranes were incubated at 4°C overnight with respective primary autoantibody in tris-buffered-saline containing 0.05% tween (TBS-T), and supplemented with 5% bovine serum albumin (BSA).

### Triton X-100 Protein Fractionation

Cells were washed with ice-cold PBS and incubated in Triton buffer (0.5% Triton X-100, 50 mM MES, 25 mM EGTA, 5 mM MgCl_2_) supplemented with 1 mM PMSF (Roth, Germany), Aprotinin, Pepstatin A (both Applichem, Germany), and Leupeptin (VWR, Germany) for 20 min on ice under continuous shaking. Thereafter, cell lysates were centrifuged at 13.000 rpm for 5 min, which leads to separation of the soluble cytosolic and insoluble cytoskeleton bound fraction. Subsequently, the pellets (insoluble fractions) were resuspended in SDS lysis buffer for Western blotting or with RIPA buffer (0.05 M Tris-HCl, 0.15 M NaCl, 0.1% SDS, 1% Nonidet P-40, 0.1 mM EDTA) for immunoprecipitation and followed by sonification. Protein concentrations were calculated as described above and equivalent amounts used for Western blotting or immunoprecipitation analyses.

### Immunoprecipitation

Following Triton X-100 mediated solubilization, cell lysates (protein amount of 1,000 μg) were precleared with 25 μl Protein A/G Agarose Beads (Santa Cruz Biotechnology, Santa Cruz, CA, USA) for 1 h at 4°C, thereafter centrifuged at 10,300 rpm for 5 min at 4°C. The supernatant (IP-lysat) was incubated with 1 μg of anti-Dsg3 antibody (Santa Cruz) or IgG control, for 3 h at 4°C with gentle rotation. The lysate was then added to 40 μl of beads and incubated overnight at 4°C under rotation. IP lysates were washed with RIPA buffer and subjected to Western blot analyses.

### *In situ* Proximity Ligation (PLA) Assay

Spatial proximities of Dsg3 and cortactin were investigated using the Duolink *in situ* kit (Olink, Bioscience) as described previously (23).

### Histology and Immunostaining

Samples were embedded in Tissue Tec (Leica Biosystems, Nussloch, Germany) and thereafter serial-sectioned at 7 μm thickness using a cryostat microtome (Cyrosstar NX70, Thermo Fisher). Hematoxylin and esoin (H.E.) staining was performed according to standard protocols (24), and mounted in DEPX (Sigma-Aldrich, St. Louis, MO, U.S.A). Images were captured using a Leica DMi8 microscope with a HC PL APO 40x/0.85 dry objective. For immunostaining, cells were seeded on coverslips and grown to confluence. After respective treatment, cell monolayers were washed with PBS and fixed with 2% paraformaldehyde in PBS for 10 min (HaCaT) or fixed with 4% paraformaldehyde in PBS for 20 min (CTTN^−/−^ and CTTN^+/+^ keratinocytes). Next, samples were rinsed several times with PBS, permeabilized with 0,1% Triton X-100 for 5 min and after final washing with PBS, blocked with 3% bovine serum albumin and 1% normal goat serum for 60 min. The primary antibodies were incubated overnight at 4°C. After washing with PBS, respective secondary antibodies were applied for 60 min at room temperature. Subsequently, coverslips were washed and mounted with 1.5% n-propyl gallate in glycerol. Images were taken with a Leica SP5 confocal microscope using a 63x/1.40 PL APO oil objective (Leica, Mannheim, Germany).

### Ca^2+^ Switch Assay

Cells were grown to confluence and, after respective treatment, incubated with 2.5 mM EGTA for 30 min (Ca^2+^-depletion), which leads to a Ca^2+^-dependent disruption of cell-cell junctions. Reformation of junctions was induced by medium change with corresponding growth medium containing 1.8 mM Ca^2+^ for 8 h (Ca^2+^ repletion).

### Dispase-Based Dissociation Assay

After incubation with test reagents, confluent cell monolayers were washed with Hank's buffered saline solution (HBSS; Sigma Aldrich) and subjected to 2.4 U/ml dispase II (Sigma- Aldrich) in HBSS for 20 min at 37°C and 5% CO_2_. After detachment of the monolayer the reaction was stopped by replacing the dispase II solution with HBSS. Defined shear stress was applied with an electrical pipette. Resulting fragments were counted using a binocular microscope (Leica, Mannheim, Germany). All independent experiments were performed in duplicates.

### Neonatal Mouse Model

The model was used as described before (25). Newborn cortactin-deficient (CTTN^−/−^) and cortactin wt (CTTN^+/+^) mice were injected intra-dermally into the back skin with a total volume of 50 μl containing 2 mg/ml AK23 without or in combination with 10 μM PP2. The area injected was marked. Twenty hours after incubation the injection site was exposed to defined mechanical stress. Skin was explanted, embedded into cryo freezing medium (Leica, Mannheim, Germany), frozen on dry ice, followed by preparation for cryo-cutting. The experimental protocol was approved by the institutional animal care and use committee of Cinvestav (IACUC), Mexico-City.

### *Ex vivo* Human Skin Model

Biopsies of healthy human skin were acquired from cadavers from the human body donor program from the institute of Anatomy and Cell Biology, Ludwig-Maximilians-Universität München, Germany. Written informed consent was given from body donors for the use of research samples. Biopsies were taken only if death occurred <24 h before arrival at the institute. From each body donor, a skin piece of approximately 5 × 5 cm size was removed from the shoulder, gently stripped off fat including excessive connective tissue. The skin was cut into 1 cm^2^ pieces and injected intra-dermally with 50 μl of the respective IgG-fraction (PV-IgG and c-IgG) with or without PP2 (10 μM), using a 30G syringe. Samples were incubated floating on DMEM at 37°C and 5% CO_2_ for 24 h. After incubation sheer stress was applied using a rubber head with equal frequency and magnitude. Treated samples were cut into two parts and processed for hematoxylin and eosin (HE) stainings and electron microscopy analyses. Blister score of the human samples was measured as described below.

### Scoring of Blister Size

Each section was evaluated and sorted into the following score system as published previously (23): absence of intraepidermal separation, score 0; cleft size covering 1–25% of total section length, score 1; cleft size between 26 and 50% of section length, score 2; cleft size between 51 and 75% section length, score 3; cleft size between 76 and 100%, score 4.

### Electron Microscopy

The injected and incubated human skin explants were cut into small pieces of approximately 2 mm in diameter and fixed with 2–5% glutaraldehyde (Sigma Aldrich). After washing in PBS, tissue samples were post-fixed with 2% osmium tetroxide (Merk Millipore), and dehydrated through a graded ethanol series followed by clearance in propylene oxide, embedding into EPON 812 (Serva Eletrophoresis GmbH, Heidelberg, Germany) and curing at 80°C for 24 h. Resulting blocks were trimmed and sectioned at 60 nm thick slices with a Reichert-Jung Ultracut E ultra-microtome using a diamond knife (DiATOME Electron Microscopy Sciences, Hatfield, PA, USA). Silver-appearing sections were placed onto a 150 mesh copper/rhodium grid (Plano GmbH, Wetzlar, Germany). Sections were then contrasted with alcoholic uranyl-acetate and lead citrate. For imaging a Libra 120 transmission electron microscope (Carl Zeiss NTS GmbH, Oberkochen, Germany) equipped with a SSCCD camera system (TRS, Olympus, Tokyo, Japan) was used.

### Statistical Analysis

Data were analyzed in Exel (Microsoft, Redmond, WA) and compared using one-way ANOVA followed by Bonferroni *post-hoc* test (for Gaussian-distributed samples) using Graphpad Prism (Graphpad Software, LaJolla, CA). Error bars represent SEM. Significance was assumed with *p* ≤ 0.05. Data are shown as mean ± SEM. Each n represent one independent experiment.

## Results

### Role of Src for Autoantibody-Induced Loss of Keratinocyte Cohesion

**Figure 1:**
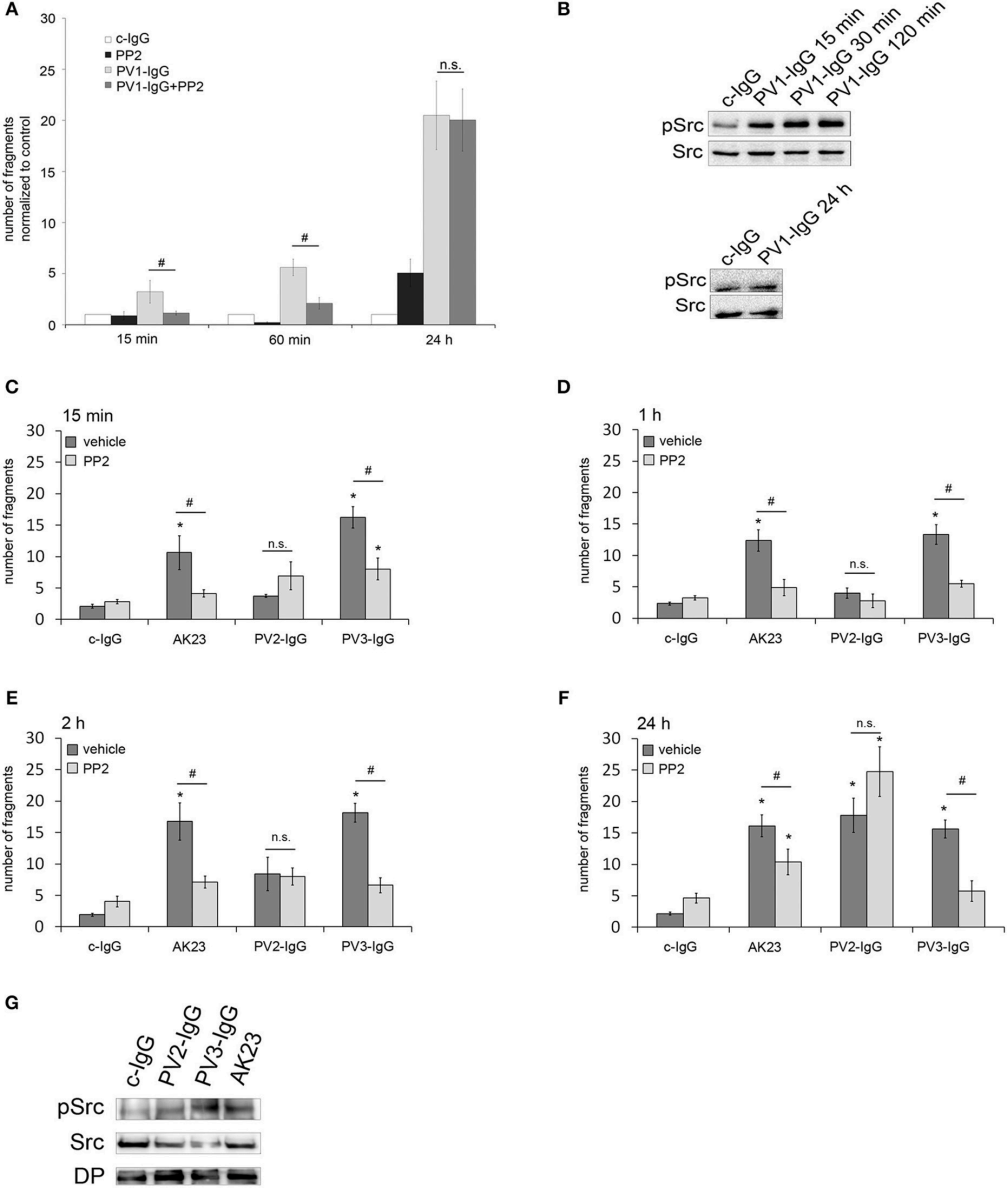
Protective effect of Src inhibition against autoantibody-induced loss of cell cohesion is variable. **(A)** HaCaT cells were incubated with PV1-IgG or with control IgG (c-IgG) and subjected to dispase-based dissociation assays. Inhibition of Src by PP2 prevented fragmentation of cell monolayers after incubation with PV1 for 15 min and 60 min but not for 24 h (*n* = 5; ^#^*p* < 0.05; *p < 0.05 vs. c-IgG). **(B)** Western blot analysis revealed that Src was phosphorylated after 15, 30, and 120 min but not after 24 h of incubation with PV1 (*n* = 3). **(C–F)** PV2- and PV3-IgG as well as AK23 were applied for several time points: 15 min **(C)**, 1 h **(D)**, 2 h **(E)**, and 24 h **(F)**, with keratinocytes being subsequently subjected to dissociation assays. Co-incubation with PP2 led to significantly reduced fragment numbers in PV3-IgG- and AK23- but not PV2-IgG-treated cells (*n* > 7; ^#^*p* < 0.05; *p < 0.05 vs. c-IgG). **(G)** Corresponding Western blot analysis for 2 h revealed that all autoantibody fractions were effective to activate Src (*n* = 3).

To investigate the role of Src for loss of cell adhesion in pemphigus, human keratinocytes (HaCaT) were incubated with PV-IgG in combination with the Src family kinase inhibitor PP2 and intercellular cohesion was measured by dispase-based dissociation assay (26). Incubation with PV-IgG from pemphigus vulgaris patient 1 (PV1-IgG) significantly increased the number of fragments in comparison to incubation with control IgG (c-IgG) for all time periods (15 min, 60 min and 24 h). Src inhibition using PP2 abrogated loss of intercellular adhesion after incubation with PV1-IgG for 15 and 60 min. In contrast, after 24 h incubation of PP2 together with PV1, no protective effect was observed (Figure 1A). After incubation of PV1-IgG for 15, 30 and 120 min, Western blot analysis showed a phosphorylation of Src at Tyr 416, which is one of the major phosphorylation sites and leads to autophosphorylation (9). In contrast, no activation of Src was detectable after 24 h of incubation, indicating that inhibiton of Src was only protective at time points when Src was activated (Figure 1B). To get more insights into the importance of Src in PV, two more IgG fractions from different pemphigus patients (PV2-IgG and PV3-IgG) as well as a monoclonal autoantibody against Dsg3 from a pemphigus mouse model (AK23) (27), were included for respective time points. In dissociation assays, PV2- and PV3-IgG as well as AK23 caused keratinocyte monolayer fragmentation after 15 min (Figure 1C), 1 h (Figure 1D), 2 h (Figure 1E), and 24 h (Figure 1F) compared to incubation with c-IgG. PV3-IgG and AK23-induced loss of cell cohesion was significantly reduced by PP2 at all time-points whereas inhibition of Src was not effective to modulate loss of adhesion caused by PV2-IgG (Figures 1C–F). Western blot analysis showed that all autoantibody fractions were effective to activate Src (Figure 1G).

**Figure 2:**
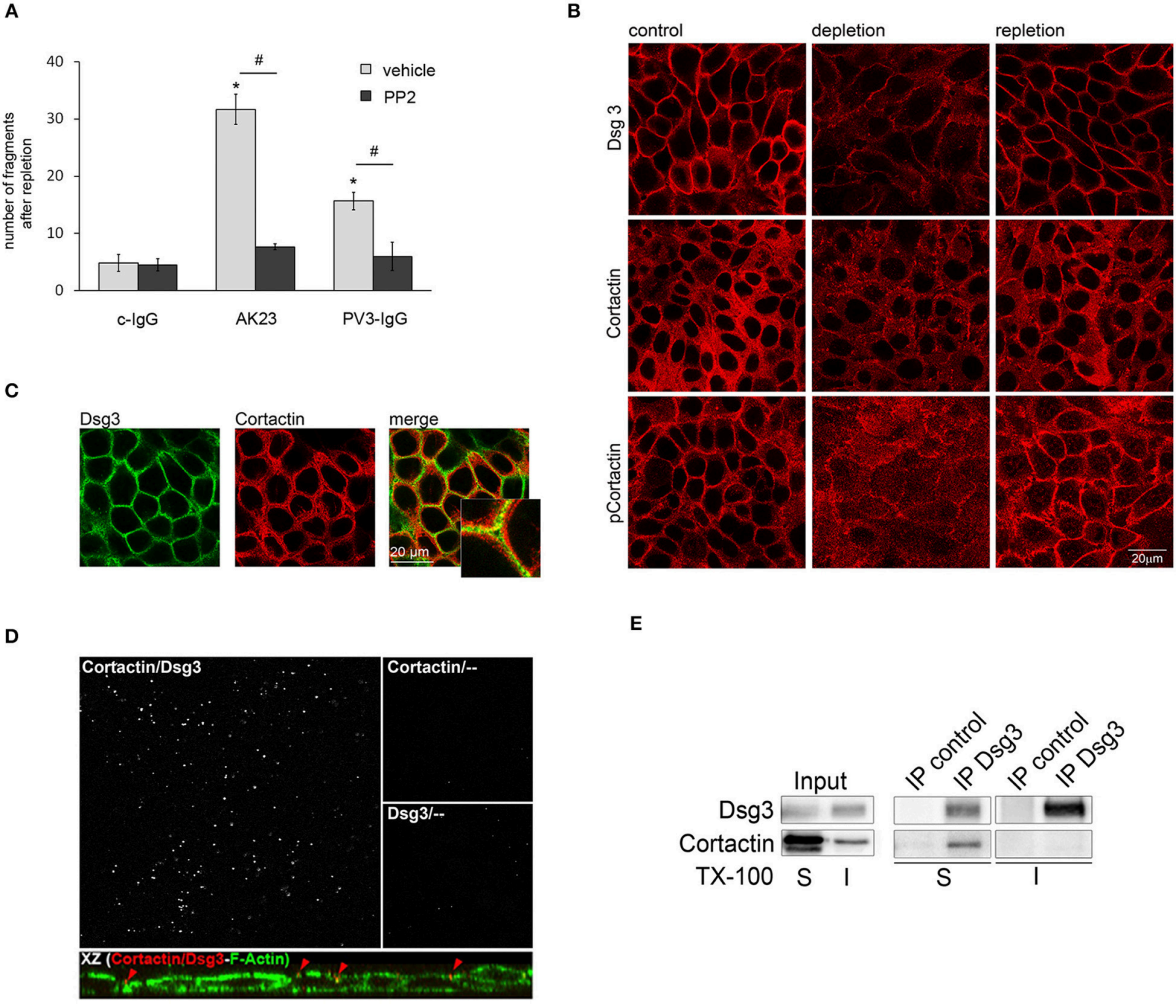
Cortactin colocalizes and interacts with Dsg3. **(A)** Under conditions of desmosome re-assembly (Ca^2+^-repletion) co-incubation with PP2 significantly reduced PV3-IgG- and AK23-induced monolayer fragmentation (*n* > 7; ^#^*p* < 0.05; *p < 0.05 vs. c-IgG). **(B)** Immunostaining revealed that under control conditions Dsg3 and cortactin as well as phosophorylated cortactin were in part localized at cell borders, which was reduced by Ca^2+^-depletion. Following Ca^2+^-repletion for 8 h, all proteins relocated along the cell-membrane (scale bar 20 μm; *n* = 4). **(C)** Under basal conditions, Dsg3 and cortactin partly co-localized at cell borders (scale bar 20 μm; insets represent 3.2x magnifications of indicated areas; *n* = 3) **(D)** Proximity ligation assay revealed co-localization of Dsg3 and cortactin close to the cell periphery. Cells were illuminated with F-actin to localize cell-structures. Incubation with cortactin or Dsg3 only served as negative control (*n* = 3). **(E)** Immunoprecipitation (IP) of Dsg3 documented a complex of cortactin within the Triton-soluble but not the -insoluble fraction (*n* = 3).

Cadherin-based adhesion is Ca^2+^-dependent. *In vitro*, the assembly of intercellular junctions in keratinocytes can be induced by a switch from low to high Ca^2+^ medium (28, 29). Vice versa, reduction of the extracellular Ca^2+^ concentration leads to translocation of cadherins from cell-cell borders to the cytosol, followed by disassembly of adherens junctions and desmosomes (28, 30). Since inhibition of Src was protective for adhesion of kerationcytes after treatment with AK23 and PV3-IgG, we investigated the role of Src for desmosomal reassembly. Ca^2+^-depleted HaCaTs were re-exposed to Ca^2+^ for 8 h in combination with c-IgG, AK23, and PV3-IgG with or without PP2 and then subjected to a dispase-based dissociation assay. Ca^2+^ repletion in combination with autoantibodies did not restore cell adhesion indicating that autoantibodies interfered with desmosome re-assembly, which likely contributes to loss of cell cohesion when autoantibodies are applied for longer periods such as shown in Figure 1. However, PP2 reduced this negative effect of both AK23 and PV3-IgG on reconstitution of cell cohesion (Figure 2A).

### The Src Target Cortactin Colocalizes and Interacts With Dsg3

The actin binding protein cortactin was identified as a major substrate of Src (17), and is proposed to be activated by Src-mediated tyrosine phosphorylation (31). Therefore, we were interested if cortactin plays a role in Src-mediated effects on desmosomal adhesion. First, we investigated the correlation of cortactin and Dsg3 in desmosomal assembly by immunofluorescence analysis under Ca^2+^ switch conditions. Under control conditions, both proteins are located at cell borders and cortactin was phosphorylated on the Src-dependent phosphorylation site Tyr 421. Depletion of Ca^2+^ resulted in Dsg3 dissociation form the cell borders, accompanied by disruption of cortactin and phospho-cortactin immunostaining. Re-administration of Ca^2+^ led to restored Dsg3, cortactin, and phospho-cortactin staining at cell-cell borders (Figure 2B). Moreover, immunostaining showed that cortactin and Dsg3 partially co-localized at the cell membrane (Figure 2C) which was validated by a proximity ligation assay (PLA), also revealing a close association with the cortical actin cytoskeleton (Figure 2D, upper images) at the cell periphery (Figure 2D, XZ presentation). Next, immunoprecipitation analysis verified that cortactin co-precipitated with Dsg3 in the triton-soluble protein pool but not in the triton-insoluble fraction (Figure 2E). This implied that cortactin may play a role in Src-mediated regulation of desmosome assembly. In contrast, phosphorylation of Pkp3 after incubation with AK23 was not detectable (Supplemental Figure 1).

### Cortactin Is Important for Src-Mediated Reconstitution of Cell Cohesion *in vitro* and in a Pemphigus Mouse Model *in vivo*

**Figure 3:**
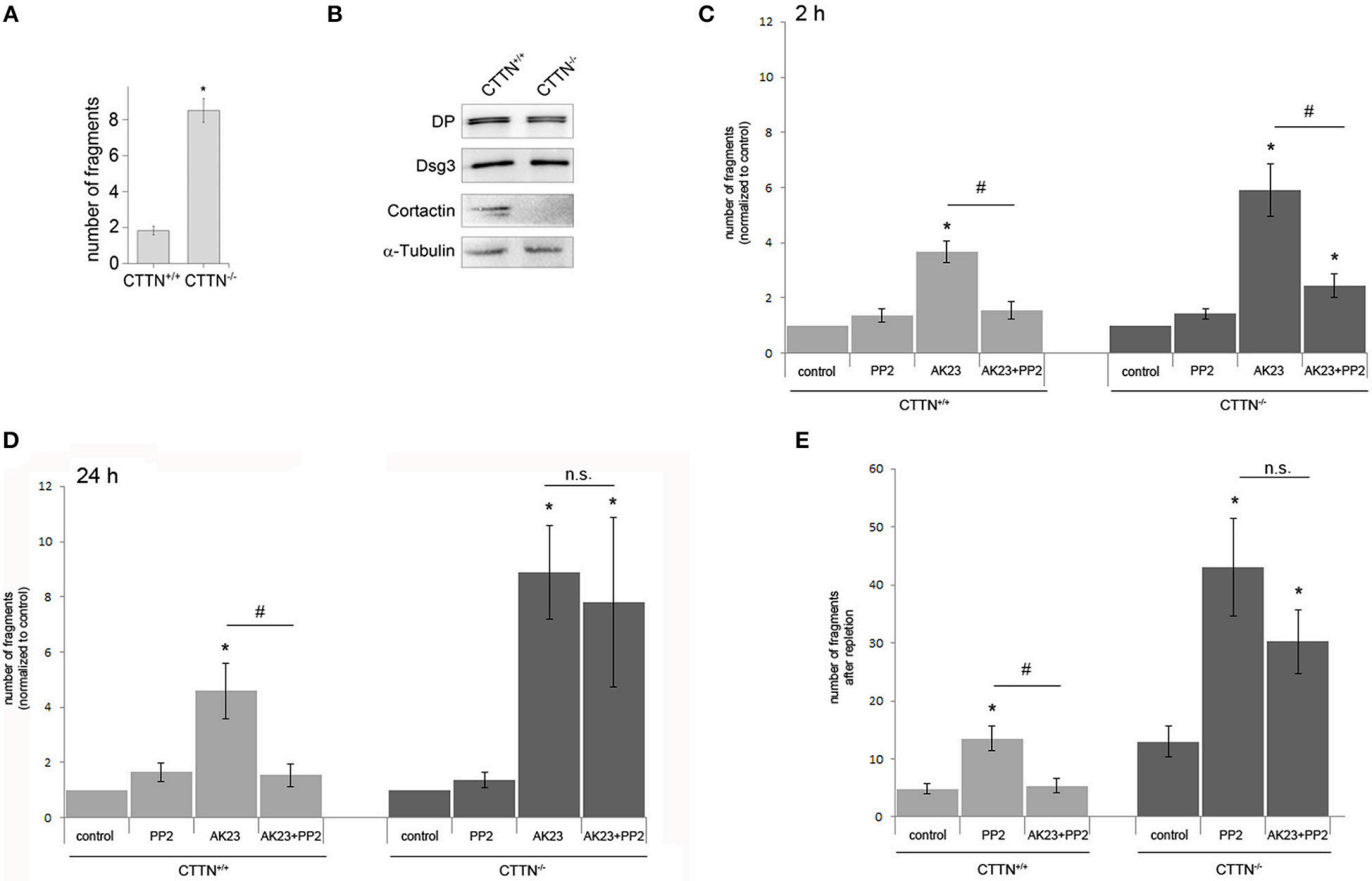
Role of cortactin in Src-mediated modulation of AK23-induced loss of cell cohesion. **(A)** Cortactin is important for cell cohesion, indicated by enhanced fragmentation of cortactin-deficient (CTTN^−/−^) cell monolayers in dispase-based dissociation assay (*n* = 5; *p < 0.05 vs. control). **(B)** Confirmation of cortactin knockout by Western blot, α-Tubulin served as loading control (*n* = 3). **(C,D)** CTTN^−/−^ and wt mouse keratinocytes exposed to a dissociation assay following either short time (2 h) or long time (24 h) incubation with AK23 (*n* > 7, ^#^*p* < 0.05; *p < 0.05 vs. control). **(E)** Reconstitution of cell adhesion measured by dissociation assay after Ca^2+^ switch in CTTN^−/−^ and wt keratinocytes in absence or presence of Src inhibitor PP2 (*n* > 7, ^#^*p* < 0.05; *p < 0.05 vs. control).

To study the role of cortactin in more detail, we isolated primary keratinocytes form newborn cortactin wild type (wt) and cortactin-deficient mice (32). Cortactin-deficient (CTTN^−/−^) mouse keratinocyte mono-layers showed significantly more fragments in a dispase-based dissociation assay, indicating that cortactin is required for cell cohesion (Figure 3A). The knockout of cortactin was verified by Western blot analysis (Figure 3B), which also showed no changes of desmosomal proteins such as desmoplakin (DP) and Dsg3 in CTTN^−/−^ cells. Next, we subjected cells to a dissociation assay after application of PP2 in combination with AK23 for 2 h (Figure 3C) or 24 h (Figure 3D). In wt mouse keratinocytes, cell adhesion was rescued by Src inhibition when AK23 was applied for 2 h or for 24 h. In contrast, in CTTN^−/−^ cells, PP2 blocked AK23-induced loss of adhesion only when AK23 was incubated for 2 h but not for 24 h. This suggested that short-term effects auf AK23 were Src- but not cortactin-dependent, whereas long-term effects at least in part required both Src and cortactin. To examine the importance of cortactin for restoring of cell adhesion, Ca^2+^-switch assay was performed under the same conditions (Figure 3E). Again, dissociation assay experiments showed that Src inhibition by PP2 was not effective to restore cell cohesion in CTTN^−/−^ cells in presence of AK23, indicating that reconstitution of cell adhesion is both Src- and cortactin-dependent.

**Figure 4:**
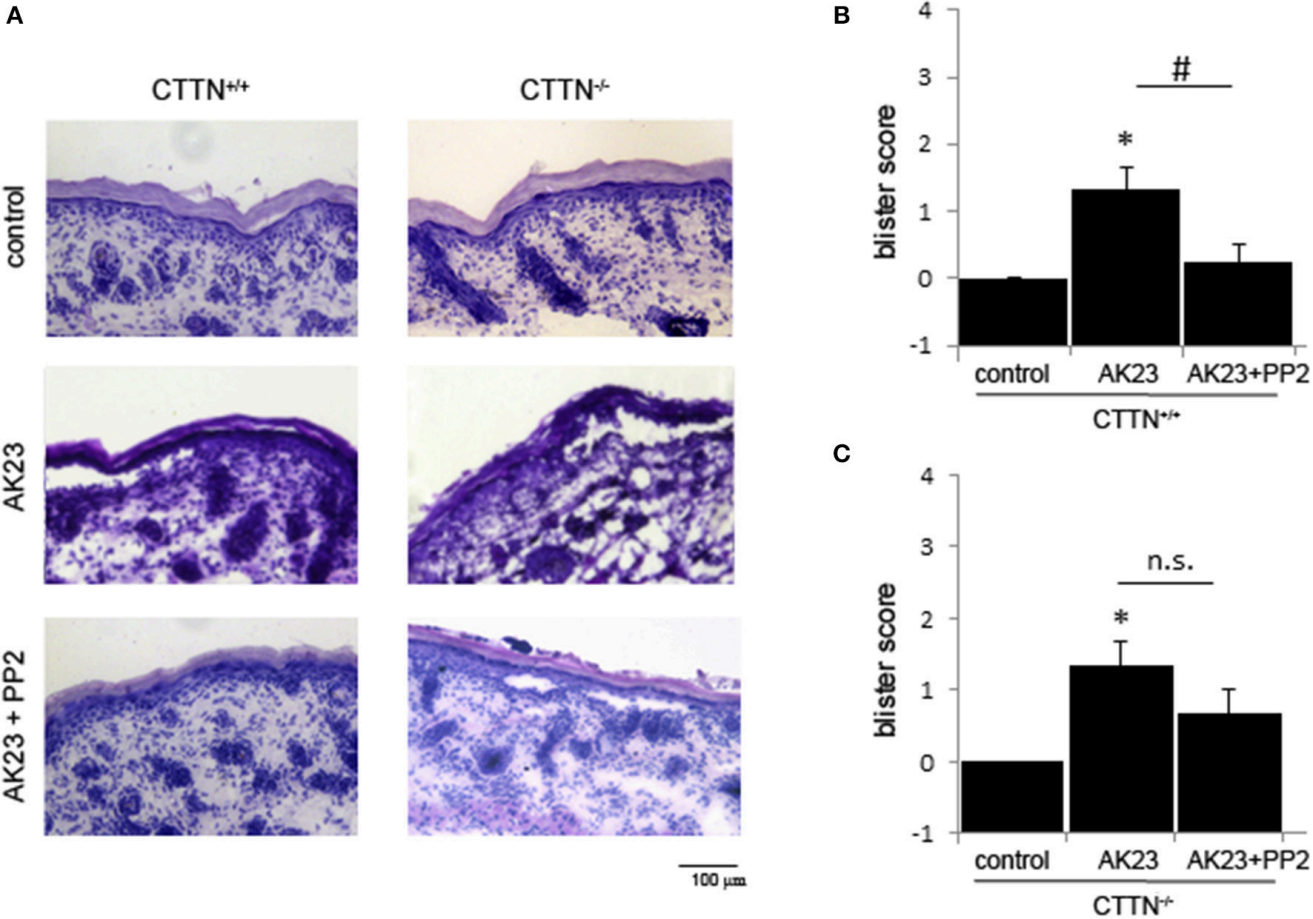
AK23-induced blister formation *in vivo* is Src- and cortactin-dependent. **(A)** H.E.-stained serial sections showed that acantholysis induced by AK23 is almost completely blocked by inhibition of Src in wt but not CTTN^−/−^ mice. Scale bar = 100 μm. **(B,C)** Blister scores for experiments described above (*n* = 3, ^#^*p* < 0.05; *p < 0.05 vs. control).

To verify these findings *in vivo*, we used the neonatal passive immuno-transfer mouse model as described previously using AK23 (23). Therefore, we injected AK23 alone and in combination with the Src-inhibitor PP2 into the skin of newborn CTTN^+/+^ and CTTN^−/−^ mice. After incubation for 24 h, defined shear stress was applied. H.E. staining of serial sections showed AK23-induced gross blistering at injection sites of both wt and cortactin-deficient pubs (Figures 4A–C). However, in wt animals co-incubation with the Src inhibitor abrogated blister formation almost completely whereas in cortactin-deficient mice, blisters were still observed. These results indicate that AK23-induced skin blistering *in vivo* is Src- and cortactin-dependent, at least, in mice.

### Inhibition of Src Was Not Protective Against PV-IgG-Induced Blistering in Human Skin *ex vivo*

**Figure 5:**
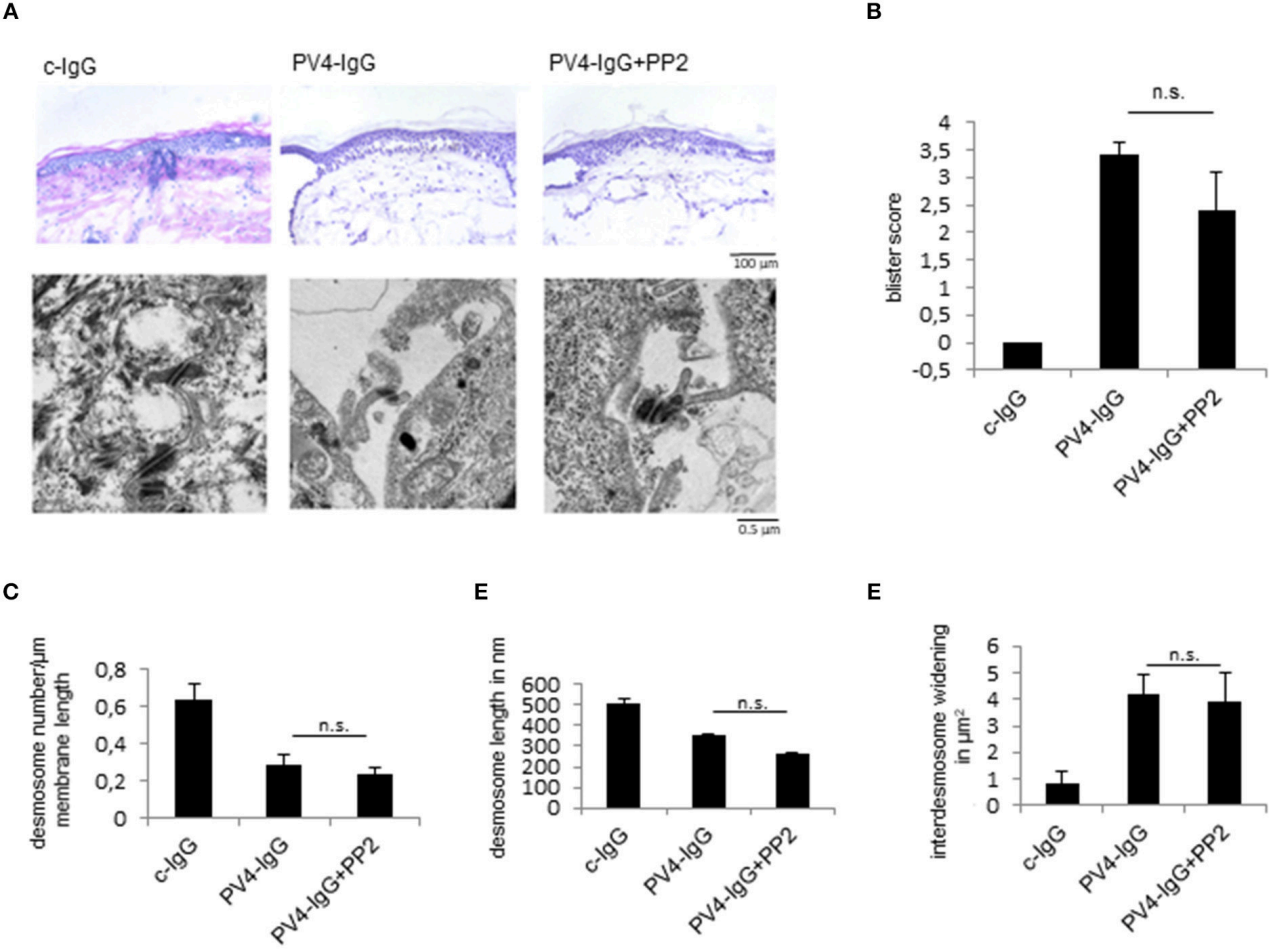
Src inhibition does not prevent PV-IgG-induced skin blistering and desmosome alterations in human skin *ex vivo*. **(A)** upper images, **(B)** H.E.-stained serial sections revealed no change in blister formation after incubation with PV4-IgG in presence of Src-inhibitor PP2 (scale bar = 100 μm). (**A**, lower images) Ultrastructural alterations of desmosomes were evaluated by transmission electron microscopy in absence or presence of PP2 (scale bar = 0.5 μm). **(C–E)** Quantification of number of the desmosomes **(C)**, desmosome length **(D)** and inter-desmosomal widening **(E)** for the conditions described above (*n* = 5).

Finally, to study the role of Src for skin blistering in intact human skin, we used a human *ex vivo* skin model as reported previously using PV-IgG (22, 33). After injection and incubation of human skin with PV4-IgG alone or in combination with the Src inhibitor PP2 for 24 h, HE-stained serial sections revealed blister formation after treatment with PV-IgG alone as well as in combination with PP2 (Figures 5A,B). Analysis of the ultrastructure showed no differences after PV-IgG incubation in conjunction with vehicle or PP2 treatment (Figure 5A, lower panel, Figures 5C–E). Under both experimental conditions, the number of desmosomes (Figure 5C) and desmosome length (Figure 5D) were reduced in comparison to control conditions and inter-desmosomal widening was observed (Figure 5E). This data show that PP2 was in the long term not protective against PV-IgG-induced skin blistering in human skin.

## Discussion

The study provides further insights into the role and mechanisms of Src-mediated epidermal blistering in pemphigus. Inhibition of Src was protective against AK23-induced skin blistering in an *in vivo* mouse model but not against PV-IgG-induced skin blistering in human epidermis. Moreover, the role of Src for loss of cell cohesion appeared to be patient-dependent and to negatively correlate at least in part with autoantibody scores against Dsg1. Src-mediated loss of keratinocyte cohesion engaged mechanisms which were both cortactin-dependent and -independent, the former of which were involved in reconstitution of keratinocyte adhesion.

### Role of Src in Pemphigus Autoantibody-Induced Skin Blistering

In accordance with the literature, PV-IgG fractions as well as AK23 led to activation of Src (4, 5, 34, 35). In this context, it was shown that PV-IgG induce Src activation early after application of autoantibodies and thus before other signaling molecules such as p38MAPK or EGFR were activated (5). The first question is by which autoantibodies Src signaling is triggered. It was shown that activation of Src was detectable only when PV-IgG fractions contained antibodies against Dsg3 but not when pemphigus foliaceus (PF) autoantibodies against Dsg1 were applied (4). Since AK23, which is specifically directed against Dsg3, was effective to activate Src it can be concluded that Dsg3 is sufficient to modulate Src signaling (4). Nevertheless, experiments using siRNA-mediated depletion of Dsg1 and Dsg3 suggest that antibodies against other targets may also be capable of activating Src (5).

Consistent with these previous findings, our data confirmed activation of Src after PV-IgG and AK23 treatment for 15 min and up to 2 h. Furthermore, loss of cell adhesion caused by AK23 was completely abolished by PP2 in cell culture *in vitro* as well as in the neonatal mouse model *in vivo*, demonstrating that Src plays an important role in loss of cell cohesion caused by autoantibodies directed against Dsg3. This supports previous findings where either broad-spectrum tyrosin kinase inhibitors or PP2 were efficient to inhibit PV-IgG-induced skin blistering in mice (35, 36).

However, inhibition of Src was not effective to consistently protect against PV-IgG-induced loss of adhesion when autoantibody fractions from different donors were applied. For one fraction, Src inhibition abrogated loss of cell adhesion only when autoantibodies were incubated short-time. Using a second fraction, no protection against loss of cell cohesion was detectable at all (27). Since all autoantibody fractions were equally effective to induce loss of cell cohesion *in vitro*, this suggests that different mechanisms maybe involved in different patients. This is supported by the observation that PP2 was not effective to inhibit PV-IgG-induced skin blistering in human skin *ex vivo*. The data presented here do not allow the conclusion that Src is not involved in human skin at all. Nevertheless, the data show that PP2 under the conditions where it is effective to block AK23-mediated blistering in mice and to prevent loss of keratinocyte cohesion in response to some patients' IgG fractions *in vitro*, is not consistently protective in intact human skin.

A limitation of the study is that the conditions used in the different experimental models do not necessarily reflect the situation in patients. Regarding the inhibition of Dsg3 with the monoclonal autoantibody AK23 derived from a pempighus mouse model (27), it should be considered that the concentration used was supraphysiologic compared to patients' autoantibody levels. This could lead to an excessive direct inhibition of Dsg3 binding and activation of signaling by Src and p38MAPK (37), both of which may not be typical in PV patients. However, in contrast to the neonatal mouse model used here, AK23 in a human skin model was not effective to induce blister formation and reduce desmosome numbers indicating that mechanisms triggered by autoantibodies against Dsg1 may be required for blister formation as well (33). Similarly, the autoantibody profiles of the PV-IgG fractions and the abundance of aDsg1 autoantibodies used in this study may at least in part explain the different roles of Src for loss of keratinocyte adhesion. PV3-IgG, the effects of which were consistently ameliorated by Src-inhibition, contained comparable amounts of autoantibodies against Dsg1 and Dsg3, whereas in all other PV-IgG fractions the relative amount of Dsg1 autoantibodies was higher. Especially, PV4-IgG used for the *ex vivo* human skin model contained excessive levels of autoantibodies against Dsg1. However, for PV2-IgG this explanation falls short because Src inhibition was not protective at all despite comparable levels of antibodies against Dsg1 and Dsg3. Since we have no information about antibodies against other antigens including desmocollin isoforms or others, we cannot rule out that such antibodies may trigger mechanisms others than Src in loss of keratinocyte adhesion.

Nevertheless, the results are in line with the hypothesis that the different clinical phenotypes of pemphigus may at least in part be determined by the mechanisms which are involved in loss of cell cohesion and engaged by antibodies targeting Dsg3 or Dsg1 (4). Moreover, the data indicate that a therapeutic paradigm to modulate Src activity alone unlikely would be effective to treat the majority of patients with epidermal blistering as this usually is associated with antibodies against Dsg1 (1). This may be related to the different mechanisms induced by autoantibodies against Dsg1 in pemphigus pathogenesis.

### Mechanisms by Which Src Regulates Cell Cohesion In Pemphigus

For some of the PV-IgG fractions such as PV1-IgG and PV2-IgG, loss of cell cohesion was clearly progressive from 15 min up to 24 h as studied in dispase assays. For other autoantibody fractions such as PV3-IgG and AK23, this effect was less pronounced which is in line with previous observations (37). This may indicate that at early time-points the underlying mechanisms may be different from later stages of adhesion loss. This is supported by the observation that short time co-incubation of AK23 with PP2 in cortactin-deficient cells can abrogate loss of cell cohesion whereas long-term incubation is not affected by Src inhibition. We conclude that short time effects of autoantibodies are in part Src- but not cortactin-dependent whereas cortactin at later stages of cell cohesion loss becomes more relevant.

We observed that autoantibodies indeed interfered with reconstitution of cell adhesion in a manner dependent on Src. Moreover, because phosphorylation of the Src substrate cortactin at cell junctions correlated with junction integrity and cortactin binds to extra-desmosomal Dsg3, we reasoned that cortactin may be involved in desmosome assembly. The interaction of cortactin with desmosomal cadherins recently has been shown for Dsg1 also (38). More detailed studies are required to elucidate how cortactin is important in this context. Here, we observed that short-time effects of autoantibodies cause loss of cell cohesion by mechanisms which do not impair formation of new desmosomes whereas at later stages, impaired reformation of junctions may contribute to adhesion loss. This can be concluded from Ca^2+^-switch assays in cortactin-deficient monolayers where Src inhibition was no longer protective against autoantibody-induced adhesion reconstitution. These data are in line with the observation that Dsg3 and Src form a complex together with E-cadherin, which appears to be involved in formation of new desmosomes (12). Since both in intact human skin *ex vivo* as well as in neonatal mouse skin *in vivo* autoantibodies were applied for 24 h, it is possible that cell cohesion in neonatal skin is more sensitive to mechanisms impairing formation of new cell contacts.

Since the pathogenesis of pemphigus appears to be complex and to include direct inhibition of Dsg3 binding as well as signaling events (2), the question arises about the role of Src and cortactin for loss of cell cohesion when compared to other mechanisms. Here we show that the short-time effects by which autoantibodies interfere with keratinocyte adhesion are at least in part dependent on Src but are independent of cortactin. Therefore, it is possible that phosphorylation of plakophilin (Pkp) 3 and its dissociation from desmosomes may be involved as has been suggested for experiments with PV-IgG (15). However, for AK23 we did not observe Pkp3 phosphorylation (Supplemental Figure 1) indicating that phosphorylation of Pkp3 can be independent of Dsg3. Aside from Src, other signaling molecules likely contribute to loss of cell cohesion in pemphigus. Electron microscopy in *ex vivo* human skin revealed that ultrastructural alterations such as loss of desmosomes, shortening of desmosomes and inter-desmososomal widening, all of which are observed in patients' lesions as well (39), were independent of Src. In contrast, these effects were shown previously to be mediated by p38MAPK (33). This is in line with the observation that pemphigus autoantibody fractions including autoantibodies targeting Dsg3 activate both Src and p38MAPK (4).

Finally, it is well-established that autoantibody titers correlate with the clinical phenotype of pemphigus (1, 3). For pemphigus autoantibodies from patients with muco-cutaneous PV or with PF, influx of Ca^2+^ and activation of ERK were also detected (4). Since Dsg1 was shown to suppress EGFR/ERK signaling by interacting with the ErbB2 binding protein Erbin (40), it is possible that autoantibodies targeting Dsg1 interfere with ERK signaling via this pathway. Taken together, we show that Src contributes to loss of cell adhesion primarily downstream of antibodies against Dsg3 in pemphigus by mechanisms which are both cortactin-dependent and –independent. The notion that actin-binding proteins such as cortactin and adducin become increasingly recognized to control desmosome function and cell behavior (12, 38), opens a new field for research on desmosome regulation.

## Author Contributions

DK, VR, EW, DE, MF, and HV-R performed experiments. DK, VR, EW, DE, and MF analyzed data. DK, EW, FV, MS, MH, RE, KR, AS, VS, and JW discussed data and interpreted results. DK and JW designed the study and wrote the manuscript.

### Conflict of Interest Statement

The authors declare that the research was conducted in the absence of any commercial or financial relationships that could be construed as a potential conflict of interest.
