# Supplementary material for: Role of Src and Cortactin in Pemphigus Skin Blistering
Source: Front Immunol. 2019 Apr 4;10:626. doi: 10.3389/fimmu.2019.00626 (PMC6461052; doi:10.3389/fimmu.2019.00626)
Supplement: Supplementary file 1 [file Image_1.pdf]

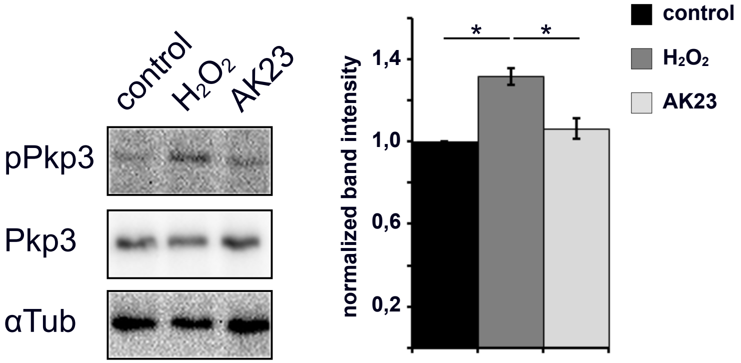

Supplemental Figure 1: Phosphorylation of Pkp3 was absent after incubation with AK23
Western blot analysis revealed that Pkp3 was not phosphorylated after incubation with AK23 for 2h. H_2O_2 served as positive control (n=4).
